# Case presentation and review of renal autotransplantation for nutcracker syndrome

**DOI:** 10.1016/j.eucr.2024.102717

**Published:** 2024-04-03

**Authors:** Kevin D. Li, Max S. Bowman, Heiko Yang, Wilson Sui, Chris Freise, Marshall Stoller

**Affiliations:** a Department of Urology, University of California San Francisco, San Francisco, CA, USA; b Department of Surgery, University of California San Francisco, San Francisco, CA, USA

## Abstract

Nutcracker Syndrome (NCS) is characterized by entrapment of the left renal vein, leading hematuria, flank pain, and proteinuria. We evaluated the efficacy of renal autotransplantation as a curative treatment for NCS through a review and case report. 55 patients from 18 studies were analyzed, with a combined 91% success rate of symptom resolution or improvement post-autotransplantation. In our case report, a 25-year-old man with severe NCS received laparoscopic nephrectomy and autotransplant, resulting in symptom resolution at 3.1 years follow up. Further research should confirm these findings and refine patient selection criteria and surgical techniques.

## 1 Introduction

Nutcracker syndrome is a rare condition characterized by the entrapment of the left renal vein (LRV) between the aorta and the superior mesenteric artery (SMA), resulting in distal dilation of the LRV.^1^ It commonly presents with a classic triad of hematuria, flank pain, and proteinuria, but symptoms also include left varicocele and nonspecific gastrointestinal derangements such as nausea and loss of appetite.^2^ Diagnosis is typically made through computed tomography (CT) or ultrasound demonstrating the “nutcracker phenomenon”, with diagnostic thresholds ranging from >50 to 80% stenosis of the LRV accompanied by symptoms typical of this syndrome.^3^ Although NCS is rare, studies suggest ∼23% of the population may have evidence of asymptomatic LRV compression on imaging.^4^^,^^5^ When symptomatic, pain associated with NCS can be debilitating and often necessitates therapeutic intervention.

The choice of treatment for nutcracker syndrome is based on the severity of the patient's symptoms. Conservative management is typically recommended for younger patients with mild to moderate symptoms. More invasive approaches include open, endovascular, and laparoscopic techniques, though there is no consensus on optimal treatment as data are limited.^6^ Endovascular approaches involve LRV stenting, with symptom resolution or improvement in >90% patients within 6 months but stent migration occurring in approximately 6.6%.^7^^,^^8^ Laparoscopic extravascular stent placement may be similarly efficacious with fewer complications.^9^ Open techniques include LRV transposition, nephropexy, gonadocaval bypass, and renal autotransplantation.^6^ LRV transposition is most commonly employed, with favorable outcomes (>86% symptom resolution at 36 months) and few postoperative complications including ileus and retroperitoneal hematoma.^7^ Limited data exist on the efficacy of renal autotransplant, with most consisting of case series. Nevertheless, the procedure appears to effectively address both the issue of SMA-aortic compression and the challenge associated with the posterior position of the left kidney.^6^

Our study presents a literature review of renal autotransplantation for nutcracker syndrome and a case presentation of a patient with severe nutcracker syndrome who was successfully treated with renal autotransplantation. The goal of this study was to demonstrate that renal autotransplantation can be curative for carefully selected patients with nutcracker syndrome.

## 2 Case presentation

We present a case of a 25-year-old male patient with a history of Crohn's disease. Over a span of four months, the patient sought care at the emergency department on 14 separate occasions, each time presenting with symptoms of acute-on-chronic left flank pain, left testicular pain, and gross hematuria.

**Fig. 1:**
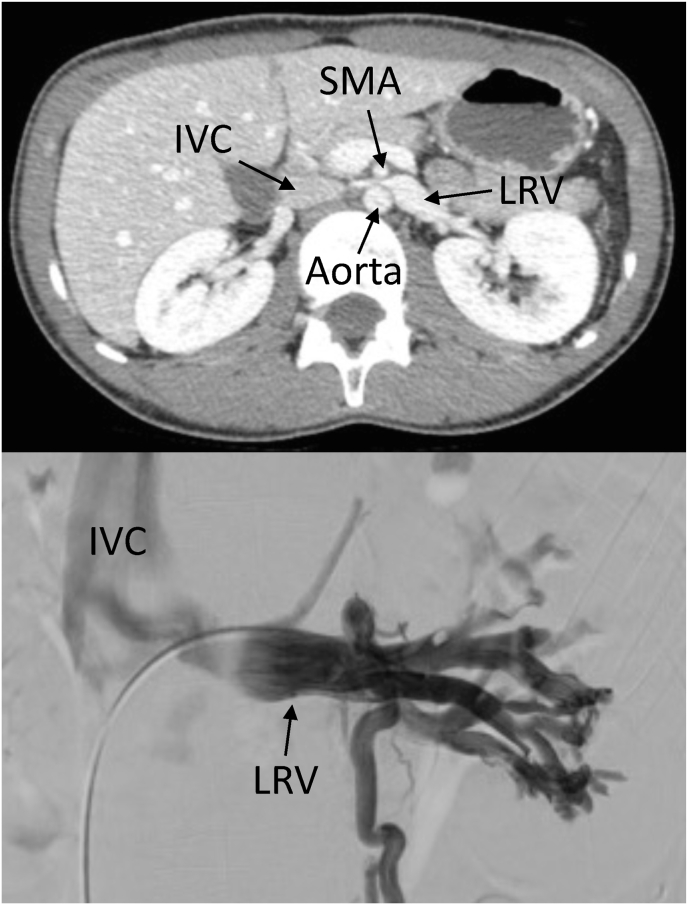
Contrast-enhanced computed tomography (CT) and renal venography showing entrapment of the left renal vein (LRV) between the aorta and superior mesenteric artery (SMA) prior to its insertion to the inferior vena cava (IVC).

Diagnostic efforts, including a contrast-enhanced computed tomography (CT) scan of the abdomen, uncovered a notable entrapment of the left renal vein between the aorta and the superior mesenteric artery (SMA) before its entry into the inferior vena cava (IVC), as illustrated in Fig. 1. Further evaluation through renal venography and manometry solidified the diagnosis.

Given the complexity of the patient's condition, multiple treatment options were considered. Renal vein transposition and stenting were deemed unsuitable due to the high risk of stent migration. Moreover, attempts to alleviate symptoms through multiple nerve blocks directed at the celiac plexus and left splanchnic nerves did not yield successful outcomes.

**Fig. 2:**
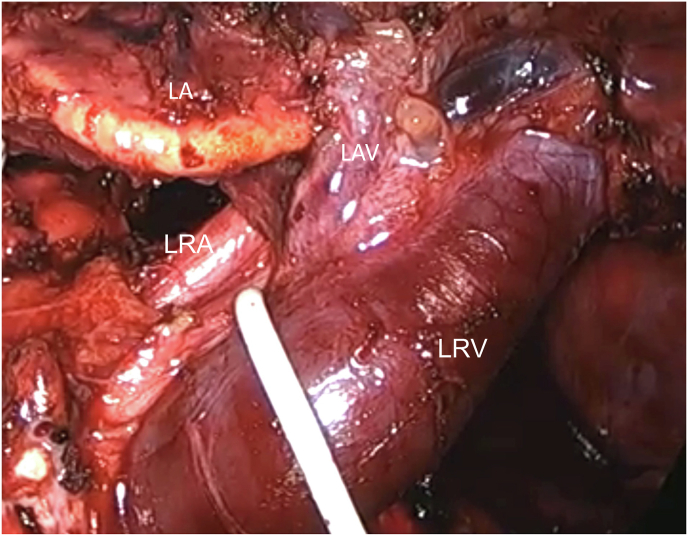
Intraoperative photo showing engorged left renal vein (LRV) along with left renal artery (LRA), left adrenal vein (LAV), and left adrenal gland (LA).

The patient underwent a laparoscopic left nephrectomy followed by autotransplantation. During the laparoscopy, we observed a markedly engorged left renal vein accompanied by numerous surrounding varices, as depicted in Fig. 2. The left gonadal vein was subsequently ligated.

For educational purposes and to provide a comprehensive view of the procedure, surgical footage of the laparoscopic left nephrectomy described above has been made available: https://ucsf.box.com/s/78co95v5rmugydrfdhznwe55jod0an49

Following the thorough mobilization of the left kidney, it was extracted through a Pfannenstiel incision. The peritoneal cavity was then meticulously closed. The resected kidney was strategically placed within the right iliac fossa. An anastomosis was formed between the renal hilum and the right iliac arteries. Additionally, a refluxing ureteral implant was introduced into the dome of the bladder to ensure urinary continuity.

The patient's postoperative course was unremarkable, and his symptoms resolved following surgery (with latest follow up at 3.1 years post-operatively).

To compare our case with existing literature, we synthesized information on patient demographics, prior interventions, surgical techniques, complications, postoperative outcomes, and follow-up durations from studies published on renal autotransplantation for nutcracker syndrome. The aggregated data are presented in Table 1.

**Table 1:** Summary of previously published studies on renal autotransplantation for nutcracker syndrome.

Study	No. of patients	Mean age	% female	Prior treatments	Nephrectomy/autotransplant approach	Post-operative outcomes	Median follow-up (months)
https://doi.org/10.1002/rcs.2508	29/32	30	97	Unknown	Robotic	Clavien-Dindo grade 1 41%, grade 2 9%, grade 3 6% (non-functional transplanted kidney secondary to torsion of vascular pedicle, chylous ascites). Complete improvement 63%, moderate improvement 16%, no improvement of pain 19%.	10.9
https://doi.org/10.6002/ect.2019.0015	4	25.7	100	LRV transposition, LRV stenting; LRV stenting, bilateral coil embolization (multiple); LRV stenting, left pelvic vein embolization; LRV transposition, LRV stenting, angioplasty of stent, embolization left gonadal vein	Open	Complete resolution of flank pain, pelvic congestion syndrome improved but not completely resolved in most patients.	7
https://pubmed.ncbi.nlm.nih.gov/20228506/	4	25.5	25	LRS transposition, LRS transposition, NA	Open	Resolution of hematuria.	4–24
https://doi.org/10.1016/j.jvsv.2019.03.003	3	26.7	100	LRV transposition; LRV bypass x2; LRV transposition	Open	Pain resolved.	12.67
https://doi.org/10.1111/j.1464-410x.1994.tb16574.x	2	32	100	NA	Open	Asymptomatic.	12
https://doi.org/10.1093/jscr/rjab580	1	22	100	NA	Robotic	Pain resolved, GI symptoms significantly improved, creatinine stable at 0.82 mg/dl.	8
https://doi.org/10.1016/j.eucr.2021.101791	1	35	100	Gonadal vein transposition, renal hilar block	Laparoscopic	Reduced left flank kidney pain.	NA
https://pubmed.ncbi.nlm.nih.gov/32106921/	1	33	100	R nephrectomy for dysplastic kidney	Laparoscopic	Free of narcotic use, excellent kidney function.	12
https://doi.org/10.1016/j.jpurol.2019.12.003	1/21	11.7	9.5	LRV transposition	Unknown	Symptoms resolved.	63.3
https://doi.org/10.1213/xaa.0000000000000848	1	38	100	Celiac plexus block	Unknown	Complete pain resolution.	6
https://doi.org/10.1016/j.euf.2018.07.019	1/10	39	0	Retrograde embolization	Robotic	Clavien-Dindo grade 2 Chyle leak managed with Sandostatin and diet. Pain and hematuria free. Creatinine and GFR stable at 0.80 and 114, respectively POD 90. Autotransplant 46% differential kidney function.	4
https://pubmed.ncbi.nlm.nih.gov/25966600/	1	22	100	NA	Laparoscopic	Azotemia postoperatively, resolved. Asymptomatic.	Unknown
https://doi.org/10.1016/j.urology.2013.10.011	1/7	52	100	LRV stenting, saphenous vein bypass	Unknown	Clavien-Dindo grade 1 (femoral neuropraxia). Unknown.	9
https://doi.org/10.1093/ndtplus/sfr152	1	19	100	Unknown	Unknown	Pain, hematuria resolved.	36
https://pubmed.ncbi.nlm.nih.gov/21646854/	1	31	0	NA	Laparoscopic	Hematuria, and left scrotum swelling resolved.	6
https://doi.org/10.1100/tsw.2011.100	1	51	0	NA	Open	Resolution of hematuria, minor analgesic requirement for pain.	1.5
https://doi.org/10.1186/1752-1947-3-82	1	21	100	NA	Laparoscopic	Resolution of hematuria, autograft normal function.	3
https://pubmed.ncbi.nlm.nih.gov/10695211/	1	27	100	NA	Open	Pain free, recently had second pregnancy.	Unknown

## 3 Discussion

Our study aimed to evaluate the effectiveness of renal autotransplantation as a curative treatment for nutcracker syndrome through a review of the literature and case presentation. We included 18 studies reporting on a total of 55 patients in our literature review. Overall, renal autotransplant was associated with favorable clinical outcomes (91% symptoms resolved or improved) and few complications. These results suggest that renal autotransplantation may be a viable option for carefully selected patients with nutcracker syndrome, especially those with severe symptoms or those who have failed or are not candidates for other treatments. However, it should be noted that further studies are needed to confirm these findings and to determine optimal patient selection criteria and surgical technique.

The pathophysiology of nutcracker syndrome (NCS) is not fully understood. Decreased perirenal fat and posterior renal ptosis with stretching of the left renal vein (LRV) over the aorta have been suggested as contributing factors.^10^^,^^11^ Recent studies have shown that abnormal branching of the superior mesenteric artery (SMA) from the aorta may also be a factor, resulting in compression of the LRV.^12^ Intermittent hematuria and left-sided varicocele are the most common clinical manifestations of NCS, with several proposed mechanisms for hematuria, including rupture of thin-walled septum between hypertensive small veins and the collecting system.^13^

Renal autotransplantation is a viable alternative for patients with NCS who are not candidates for endovascular intervention or LRV transposition, who wish to avoid the potential complications of endovascular stenting, or in those who desire a more definitive cure. In our review, two Clavien-Dindo grade 3 complications occurred, including torsion of the vascular pedicle leading to loss of the transplanted kidney and chylous ascites requiring percutaneous drainage. The authors noted that the former complication likely arose from intraperitoneal kidney placement and suggested retroperitoneal placement to reduce the risk of torsion.^14^ No studies have been conducted to determine the optimal surgical approach for renal autotransplantation, likely due to considerations such as patient expectations, physician experience, and institutional resources.

Campsen et al. (2021)^15^ introduced an algorithm for classifying the severity of nutcracker syndrome and determining appropriate treatment. Severity is classified into four levels, with level 1 indicating asymptomatic patients with imaging findings only, level 2 with accompanying pelvic pain or congestion, level 3 with changes on renal venogram (pressures >2 mmHg and gonadal vein enlargement with collateralization), and level 4 with a history of prior surgeries, insults to the kidney, or alternative anatomy (such as retroaortic vein or multiple arteries). Campsen et al. propose performing a renal hilar block by administering local anesthetic near the ipsilateral renal artery and evaluating the patient's pain response. Pain reduction indicates a suitable candidate for autotransplantation and thus this can be useful for patient selection.

Our study has limitations that should be acknowledged. Firstly, we only included 18 studies reporting on 55 total patients, which may limit generalizability of our findings. Additionally, there was variability in techniques used for autotransplantation, and some data were not available, introducing potential biases in our analysis. It is important to note that a significant proportion (>50%) of the sample was derived from Mejia et al., which included both patients with neurogenic bladder dysfunction and loin-pain hematuria syndrome. This could account for the observed 19% of patients who did not experience improvement in pain symptoms.

## 4 Conclusion

We demonstrated that laparoscopic left nephrectomy with autotransplantation can be successful in treating debilitating symptoms caused by Nutcracker Syndrome when other treatment options are not feasible. This operation is well tolerated and successful in appropriately selected patients. Further studies are warranted to determine long-term outcomes and to identify optimal candidates for this procedure.

## CRediT authorship contribution statement

**Kevin D. Li:** Data curation, Formal analysis, Investigation, Methodology, Visualization, Writing – original draft, Writing – review & editing. **Max S. Bowman:** Conceptualization, Resources, Writing – original draft, Writing – review & editing. **Heiko Yang:** Conceptualization, Resources, Supervision, Writing – review & editing. **Wilson Sui:** Conceptualization, Investigation, Supervision, Writing – review & editing. **Chris Freise:** Conceptualization, Data curation, Resources, Supervision, Writing – review & editing. **Marshall Stoller:** Conceptualization, Data curation, Investigation, Methodology, Project administration, Resources, Supervision, Writing – original draft, Writing – review & editing.
